# Supplementary figures and images for: Data-driven approach for the delineation of the irritative zone in epilepsy in MEG
Source: PLoS One. 2022 Oct 25;17(10):e0275063. doi: 10.1371/journal.pone.0275063 (PMC9595543; doi:10.1371/journal.pone.0275063)

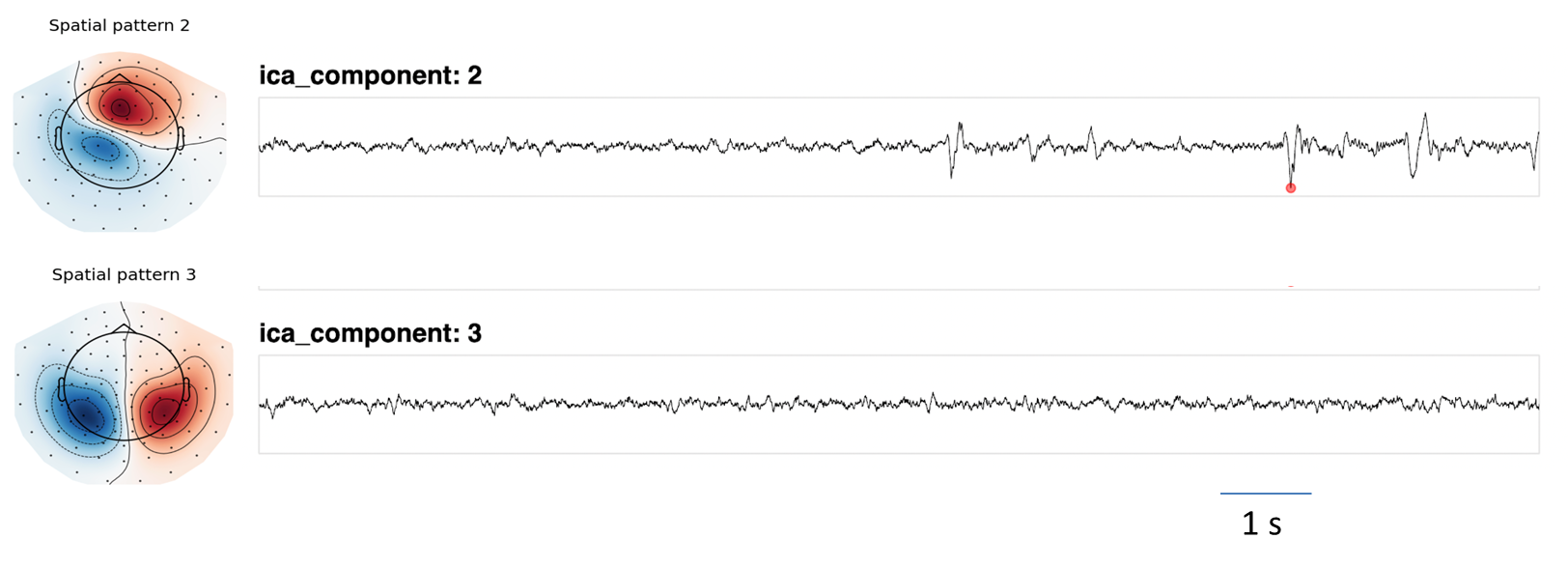

Supplement: S1 Fig — In case #4, the ICA decomposition of magnetometers provided components 3 and 4, both featuring a dipolar spatial pattern (GOF ICA3 = .96, GOF ICA4 = 0.98). However, only ICA component 2 presented spike-like events in the temporal pattern, which was reflected in a Kurtosis value of 5.6, while the Kurtosis for ICA component 3 as 0.7. The code to reproduce this image is published at https://github.com/MEG-SPIKES/aspire-alphacsc-epilepsy-MEG/blob/main/analysis/05_revision.ipynb. (TIFF) [file pone.0275063.s001.tiff]

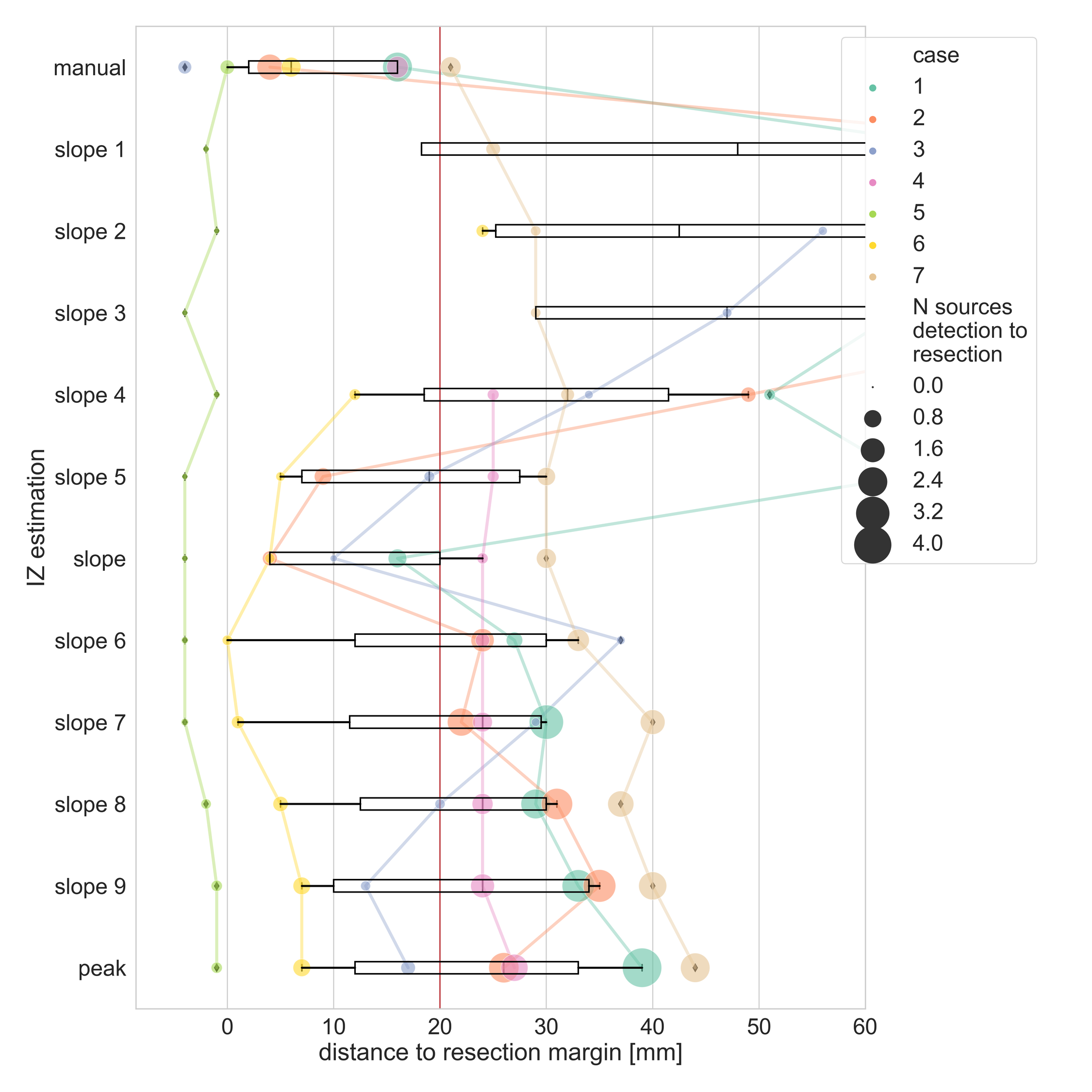

Supplement: S2 Fig — Slope 1–5 refers to latencies between the take-off and the 50% slope. Slope 6–9 refers to latencies between the 50% slope and the peak. The latencies were individually selected for each cluster because of the different length of the ascending slopes. The code to reproduce this image is published at https://github.com/MEG-SPIKES/aspire-alphacsc-epilepsy-MEG/blob/main/analysis/05_revision.ipynb. (TIFF) [file pone.0275063.s002.tiff]

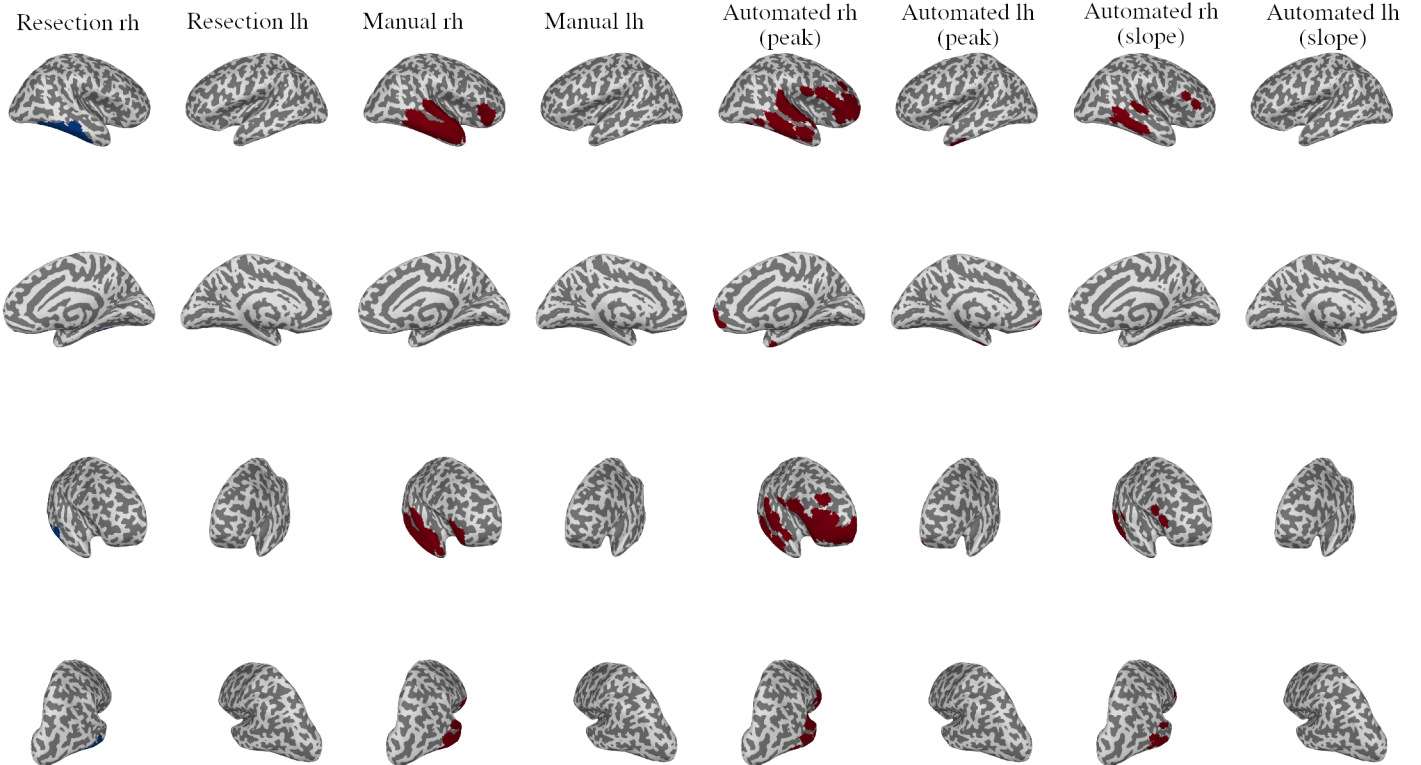

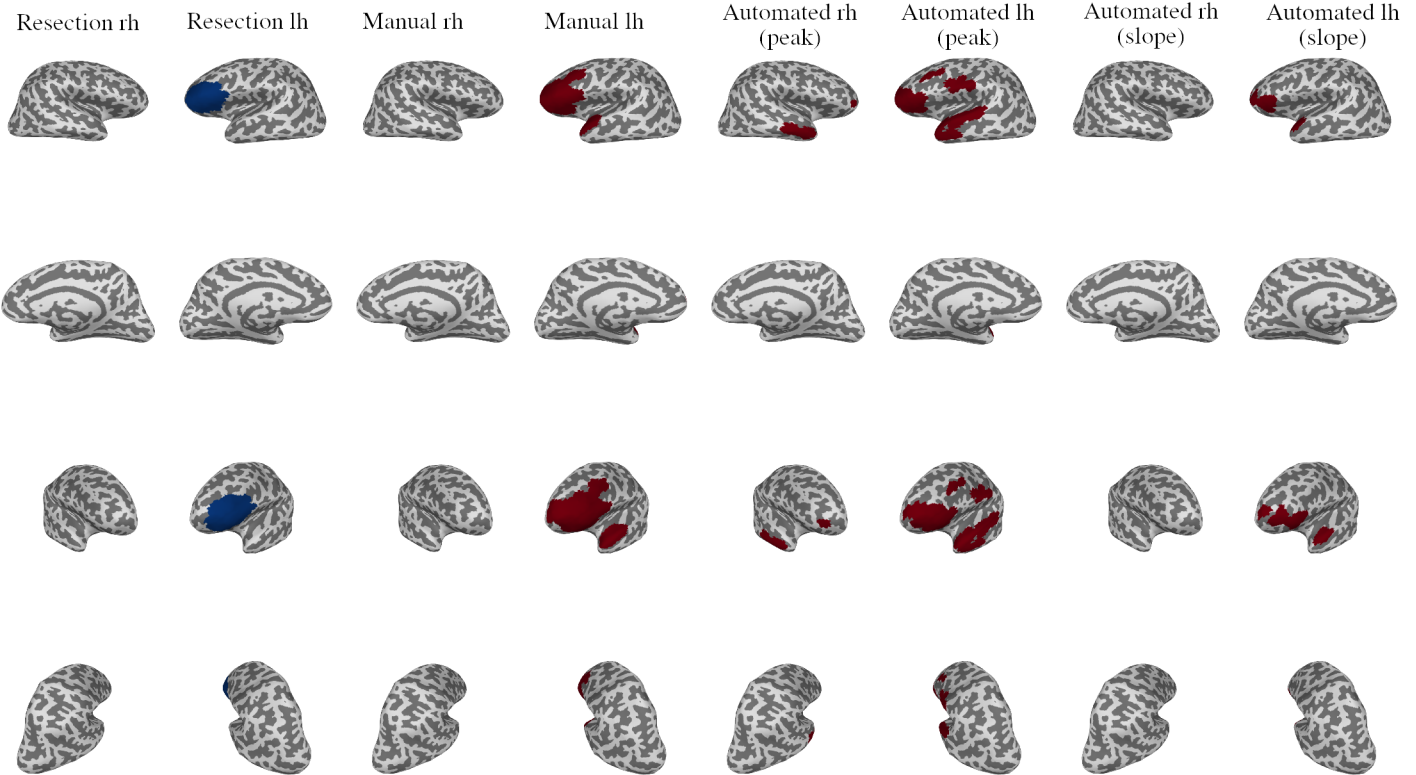

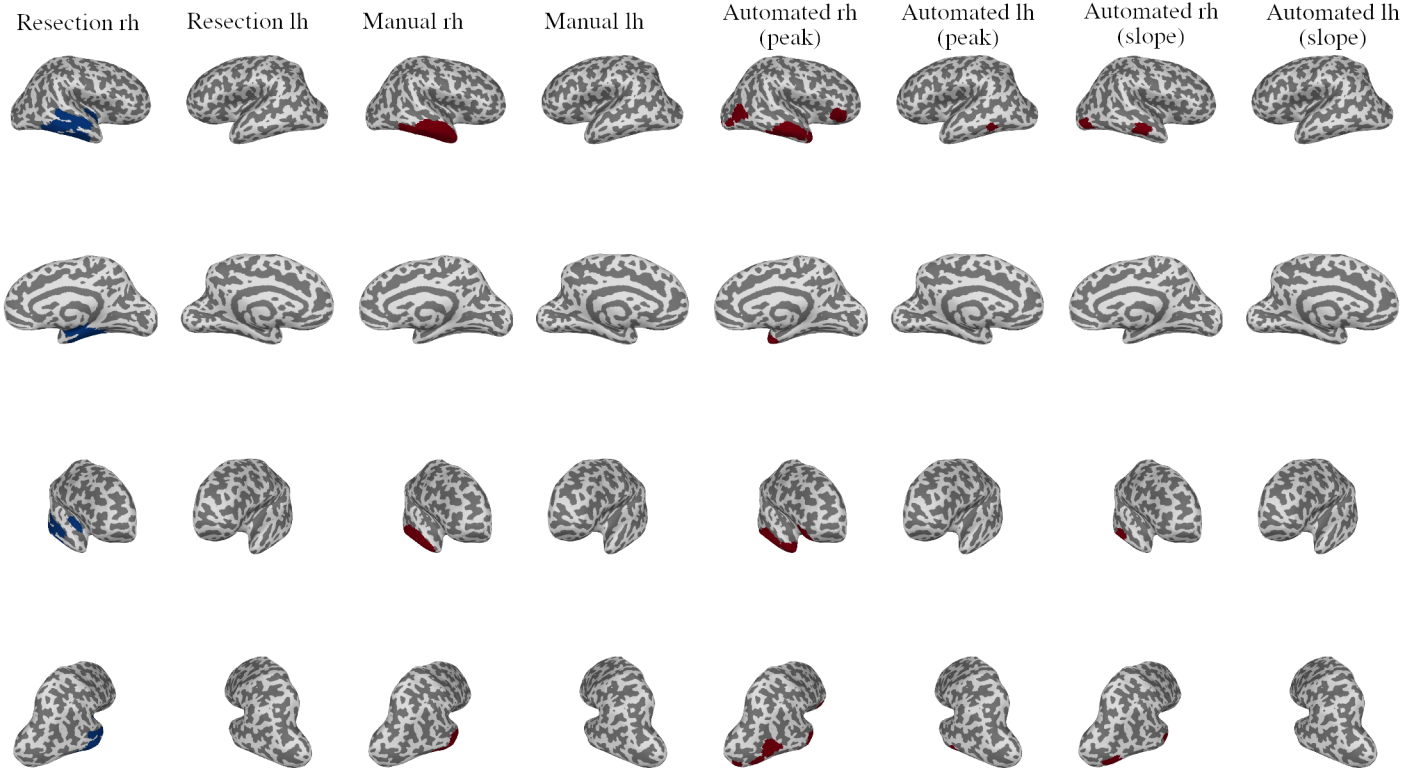

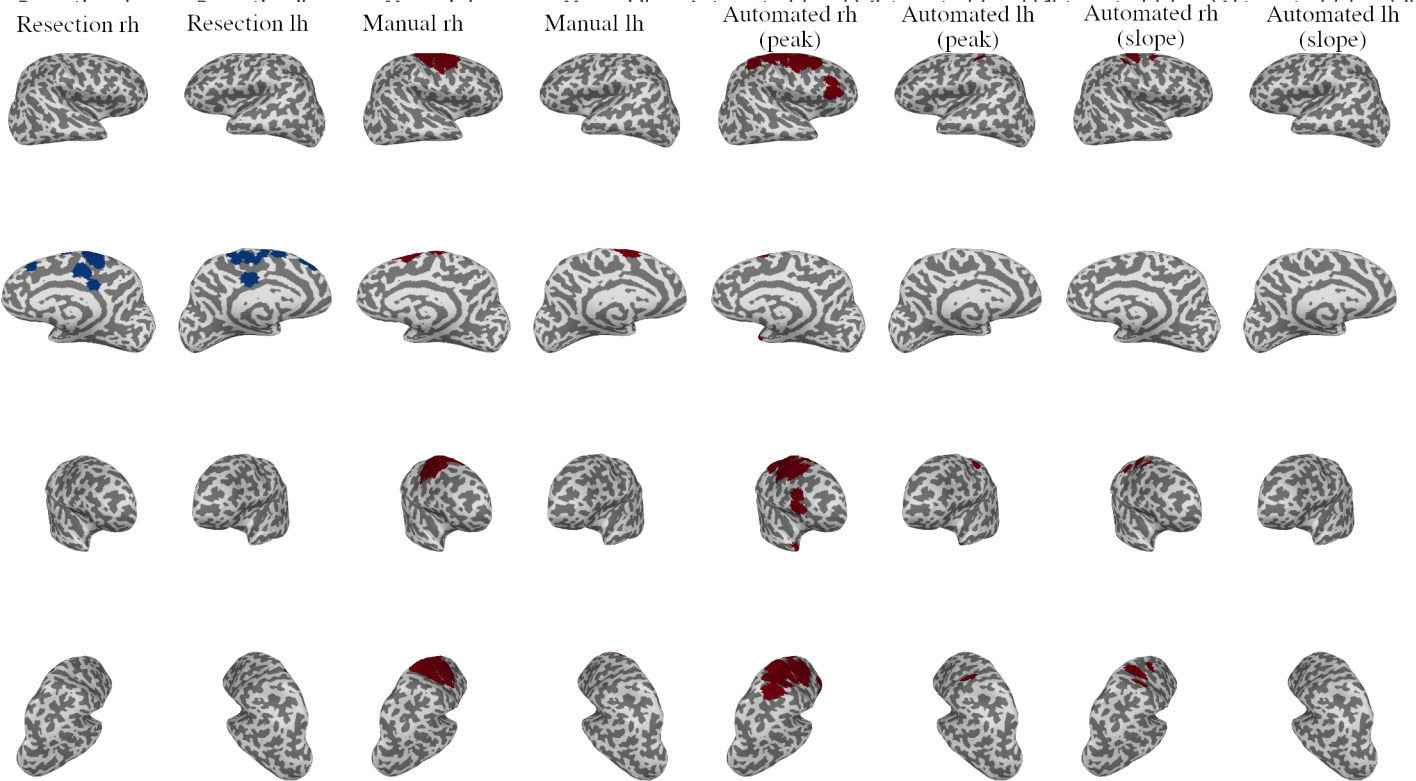

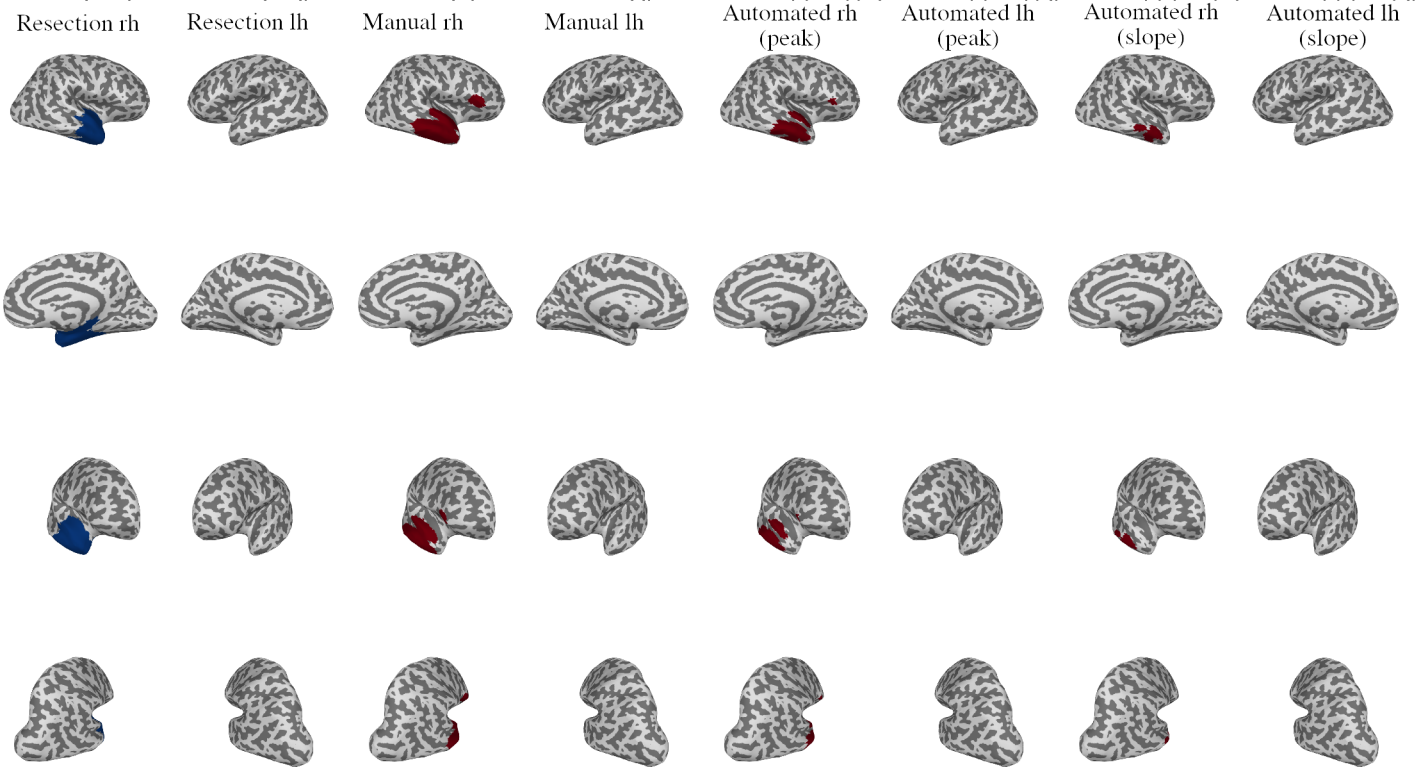

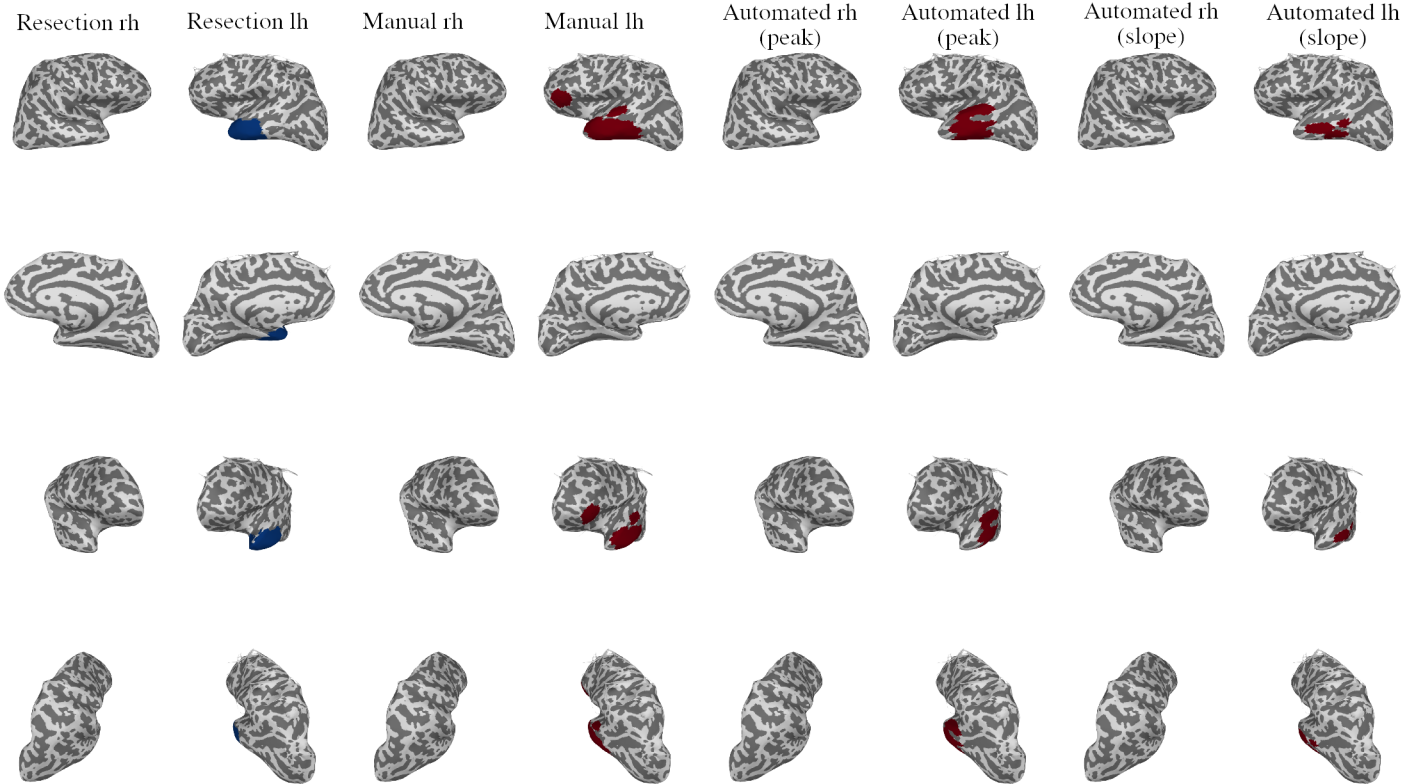

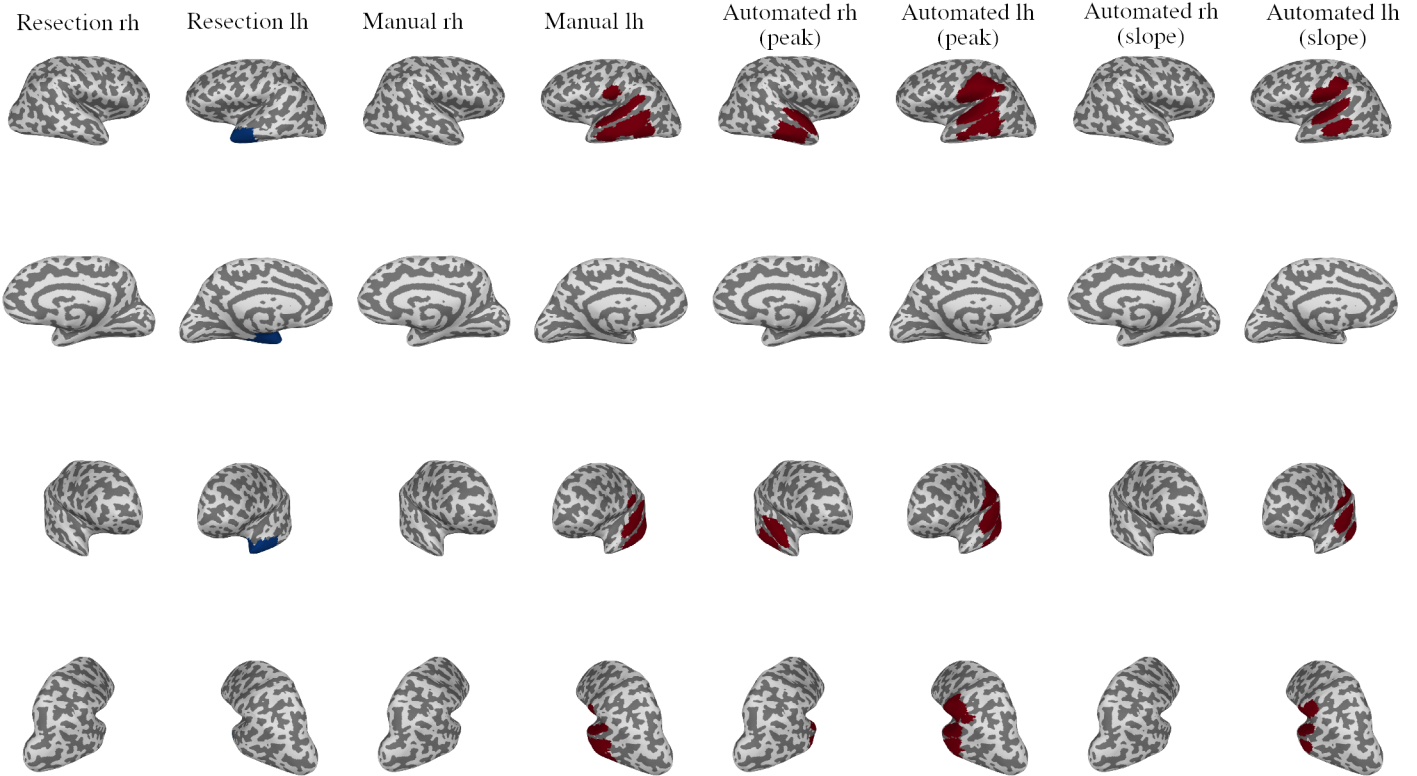

Supplement: S3 Fig — Pages 1–7 correspond to patient 1–7 in Table 1. (PDF) [file pone.0275063.s003.pdf]
