# Supplementary material for: Data-driven approach for the delineation of the irritative zone in epilepsy in MEG
Source: PLoS One. 2022 Oct 25;17(10):e0275063. doi: 10.1371/journal.pone.0275063 (PMC9595543; doi:10.1371/journal.pone.0275063)
Supplement: Attachment — Submitted filename: Response to Reviewers.docx [file pone.0275063.s004.docx]

Response to Reviewers

We thank the reviewers for their valuable comments.

We answer here point by point to the reviewers comments and provide the link to changes in the manuscript.

1. In the discussion section, the authors should justify scientific value or practical advantages of their approach.

RESPONSE: we now improved the discussion section:

At the beginning:

“ Our approach provided an estimation of the irritative zone consistent with the visual inspection of expert observers. Therefore our results support the standardization of the analysis of interictal epileptiform activity in MEG data. “

In the explanation of our pipeline, as an optimization to MEG data of an algorithmic strategy applied to spike sorting in microelectrodes recordings:

“Our approach follows a procedure similar to spike sorting in microelectrodes recordings (Yger et al. 2018), where detected neuronal spike peaks are used to build spatio-temporal templates by density-based clustering. Clustering epilpeptic spikes in MEG recording represents a more challenging problem, given the lower number of events and their signal to noise ratio (SNR). On the other hand, given the biophysical relation between source and sensor space in MEG data, we optimized this scheme for the delineation of the irritative zone in non-invasive human epilepsy data. “

We also discussed practical advantages of successful automated IED detection for clinicians:

“ the proposed approach quickly provides neurologists with candidate clusters, which can be inspected and possibly contribute to clarify the structure of the epileptic network. “

We discuss how the localization of the averaged cluster improves the SNR of the spatiotemporal profile of IED, and therefore allow reliable source reconstruction at latencies preceding the spike peak, which is crucial in case of spike propagation:

“The identification of epileptiform activity is a crucial step to anatomically target spike generators. …. Enhancing the SNR of repeating patterns, we might provide a finer localization of the event onset.”

2. Based on the study from Jas et al. (2017), the spatiotemporal clustering method used in this study (convolutional sparse coding, CSC) could be sensitive to artefacts that are common in MEG recordings of active epilepsy patients. How are those artefacts handled in this study?

RESPONSE: we selected 20 minutes of sleep (section 2.2) to minimize the amount of artifacts, and visually inspected the data removing noisy segments (2,3). In the first stage of the algorithm, we validate the identified peaks by selecting ICA components with high localization accuracy, mimicking a bipolar spatial pattern. If the input to the CSC stage would still contain some artifact, the convolutional sparse coding is based on alpha stable (heavy tailed) noise distributions, which makes it by construction robust against bursty events. Therefore, our pipeline is robust in terms of artifact rejection.We now improve our discussion session:

“The data feeded into the pipeline were visually screened to minimize the presence of artifact. The peak detection wa is performed on ICA components mimicking sources with high kurtosis. Searching for peaks in source space rather than in sensor space signal improves signal-to-noise ratio (SNR) and is more robust to artifacts. As an advancement on [(Ossadtchi et al. 2004)](https://www.zotero.org/google-docs/?broken=aMF8Cc), we did not cluster separately with respect to spatial and temporal source features of each single event, but we rather exploited the consistency of the spatiotemporal pattern of multiple events, which led to the estimation of data-driven ‘atoms’. Convolutional sparse coding is based on alpha stable heavy tailed noise distributions, which makes it robust against bursty events. The ‘atoms’ defined the library of learned repeating events, with each ‘atom’ representing the center of each cluster in feature space. “

3. After clustering IED events in magnetometers and gradiometers separately, were the same events combined/aligned? If so, how were the same events identified separately aligned based on peak time point? How did this affect the MNE final solution?

RESPONSE: We considered magnetometers and gradiometers as independent measurements. More specifically, clusters of events were averaged and source reconstructed separately for each sensor type. The information from different sensors was merged only in the source space (section 2.6.3). We did not inspect the alignment of magnetometers and gradiometers, as we are planning to investigate this issue in a follow-up study.

We now stress this in the methods section 2.6.3: “ Atoms resulting from gradiometers and magnetometers were treated as independent contributions. ”

4. In Page 6 section 2.6 line 3, Yger et al. 2018 (typo as well i.g should be i.e.), how does the Author's approach actually differ from the Yger approach that is referred to as being "conceptually related"? Please clarify this point.

RESPONSE: We thank the reviewers for the opportunity to clarify this aspect. We initially tested SpyKING CIRCUS, from Yger et al. 2018, which included the following main steps:

1.	Threshold-based peak detection

2.	Templates’ built by density-based clustering on detected peaks

3.	Templates’ matching of detected events

MEG data represent a more challenging problem in terms of SNR and number of events. At the same time, the multivariate nature of MEG data biophysical relation between source and sensor space allowed us to improve on the following aspects:

1.	Unlike SC, spike detection in our pipeline involves ICA decomposition, ICA components selection, peaks’ detection on selected ICA components and source reconstruction of the detected events (Ossadtchi et al., 2004) (section 2.6.1). All these steps are necessary to avoid selecting unreliable events before the spatio-temporal clustering.

2.	Spatio-temporal clustering is performed using convolutional sparse coding (La Tour et al., 2018) instead of density-based clustering (Rodriguez & Laio, 2014) as in SpyKING CIRCUS (section 2.6.2).

3.	Templates’ matching step from SC is replaced in our pipeline with an alignment of ICA-based detections using the activation vector (*z_k_*) from convolutional sparse coding (section 2.6.2).

For clarity, we now removed this sentence from the methods section and moved this message to the second paragraph of our Discussion:

“Our approach follows a procedure similar to spike sorting in microelectrodes recordings (Yger et al. 2018), where detected neuronal spike peaks are used to build spatio-temporal templates by density-based clustering. Clustering epilpeptic spikes in MEG recording represents a more challenging problem, given the lower number of events and their signal to noise ratio (SNR). On the other hand, given the biophysical relation between source and sensor space in MEG data, we optimized this scheme for the delineation of the irritative zone in non-invasive human epilepsy data. “

5. In Page 6 section 2.6.1 components selection, do authors quantify the variance of components selection between raters, as the selection process could be subjective and bias may be introduced?

RESPONSE: the ICA component selection was run automatically following the described criteria for goodness-of-fit and Kurtosis. No human rater was involved.

We highlight this in text in the 2.6.1 - Components selection: “ ICA components meeting these criteria were automatically selected for the following analysis. “

6. The spatiotemporal clustering method used in this study (convolutional sparse coding, CSC, La Tour et al., 2018) has been accepted by NeurIPS 2018. Please cite the conference paper, instead of BioRxiv preprint.

RESPONSE: we now updated the reference. We thank the reviewer for the notification.

7. Can the Authors be less subjective in their description of 'peakiness' for their ICA characterisation. It is not clear to the reader how this looks when differentiating between less peaky and more peaky components. An image might help.

RESPONSE: We measured the peakyness through the Kurtosis of the time series of ICA components (Ossadtchi et al. 2004). Time series characterized by high Kurtosis are more likely to carry IED. We provide here an example of peaky and non peaky ICA time trend. We now include this example as supplementary material Figure S1, and refer to it in section 2.6.1 - Components selection in the manuscript:

“high Kurtosis values (1 to 10, see also Figure S1), “

*Figure S1. Selection of “Peaky” ICA components. In case #4, the ICA decomposition of magnetometers provided components 3 and 4, both featuring a dipolar spatial pattern (GOF ICA3 = .96 , GOF ICA4 = 0.98 ). However, only ICA component 2 presented spike-like events in the temporal pattern, which was reflected in a Kurtosis value of 5.6, while the Kurtosis for ICA component 3 as 0.7.The code to reproduce this image is published at* [*https://github.com/MEG-SPIKES/aspire-alphacsc-epilepsy-MEG/blob/main/analysis/05_revision.ipynb*](https://github.com/MEG-SPIKES/aspire-alphacsc-epilepsy-MEG/blob/main/analysis/05_revision.ipynb)

[Ossadtchi, A., S. Baillet, J. C. Mosher, D. Thyerlei, W. Sutherling, and R. M. Leahy. 2004. “Automated Interictal Spike Detection and Source Localization in Magnetoencephalography Using Independent Components Analysis and Spatio-Temporal Clustering.” *Clinical Neurophysiology* 115 (3): 508–22. https://doi.org/10.1016/j.clinph.2003.10.036.](https://www.zotero.org/google-docs/?Q9OUL6)

8. With means at VIS 8.4mm, SLOPE 19.7mm, PEAK 30.7mm from the resection margins, the Authors conclude that "consideration of source spread at the ascending slope provided an IZ closer to RA (resection area) and resembled the analysis of the expert observer". I think the term 'resemble' is an overstatement as there is a clear gap between the VIS and the automated results.

RESPONSE: Following the reviewer suggestion we now tone down the terms “resemble” in the abstract of the manuscript: “The consideration of the source spread at the ascending slope provided an IZ closer to RA and to the analysis of an expert observer”

9. Further to Point 7, to argue that results would be closer to the accuracy of the VIS results closer to the take-off is conjectural. Why not run a further analysis closer to take-off (eg. half-way between take-off and the 50% rising phase?

RESPONSE: We share the reviewer’s interest in the information contained in the take-off latency and we tested the possibility to extract valuable information from earlier latencies of the ascending slope. We illustrate the results of this analysis in the figure, where we show stable SNR for latencies selected at 50% slope and later, while between take off and 50% slope the reconstruction does not provide valuable clinical information. We find our results in line with Plummer et al., 2019.

We provide the outcome of our analysis in Figure S2 in Supplementary Material, and refer to it in the Methods, section 2.6.3:

“An activation map was computed for each atom at two latencies identified as PEAK, i.e. the latency of maximum amplitude of the sharp deflection of the spike, and SLOPE, identified as the latency preceding the PEAK where the activity is still above baseline and the spatial pattern provides a distinct focus (see also supplementary material, figure S2)”

[Plummer, Chris, Simon J. Vogrin, William P. Woods, Michael A. Murphy, Mark J. Cook, and David T.J. Liley. 2019. “Interictal and Ictal Source Localization for Epilepsy Surgery Using High-Density EEG with MEG: A Prospective Long-Term Study.” *Brain* 142 (4): 932–51. https://doi.org/10.1093/brain/awz015.](https://www.zotero.org/google-docs/?Q9OUL6)

*Figure S2. Distance from the resection margin from take-off to peak of the averaged clusters. Slope 1-5 refers to latencies between the take-off and the 50% slope. Slope 6-9 refers to latencies between the 50% slope and the peak. The latencies were individually selected for each cluster because of the different length of the ascending slopes. The code to reproduce this image is published at* [*https://github.com/MEG-SPIKES/aspire-alphacsc-epilepsy-MEG/blob/main/analysis/05_revision.ipynb*](https://github.com/MEG-SPIKES/aspire-alphacsc-epilepsy-MEG/blob/main/analysis/05_revision.ipynb)

10. And further to Points 7 and 8, the logic of 'source spread' as an explanation for the differences between VIS and Automated appears to be contradicted by the fact that VIS results appeared to be based on spike peaks (Section 2.6.3, line 5). This needs to be addressed in the Discussion.

RESPONSE: The reviewer is correct to highlight that VIS results are based on spike peaks. However, VIS spike analysis is based on the localization of a choice of selected events, which in the judgement of visual observer were representative of the clinical case. Our automated analysis follows selection criteria which are dictated by a set of described mathematical assumptions, which were equal among the considered dataset. This led to a larger spread of the estimated irritative zone of PEAK compared to VIS. However, the advantage of the automated procedure is that, while the choice of events will might be suboptimal with respect to VIS analysis, the clustering provides the opportunity to improve the SNR at the level of the ascending slope, which results in a comparable performance to the VIS. We now changed our phrasing at the end of the Results section:

“However, only when considering the source activation at the spike onset, the performance of the automated detection is similar to the expert observer. ”

And added to the Discussion section:

“In this context, an automated approach based on peak analysis might overestimate the extension of the irritative zone. Clustering events allows access to source activity at latencies preceding the spike peak with higher SNR. The proposed approach quickly provides neurologists with the activation profile of candidate clusters, which can be inspected and possibly contribute to clarify the structure of the epileptic network.”

11. Only < 2 mins recording for the automated analysis. How long were the full recordings? How can the Authors be certain that this very short period was representative of the entire recording?

RESPONSE: In section 2.3 we write “We selected a 20 minutes recording with the highest number of visually marked spikes for each patient. MEG data were visually inspected and noisy segments were excluded for the automated spike detection pipeline (< 2 minutes out of 20-minutes recording). “The full recording lasted around 1 - 2 hours, but we focussed on the 20 minutes segment with the patients sleeping, minimal presence of movement artifact, and largest number of annotated visually marked IED. These 20 minutes were the most representative for the analysis of the irritative zone in each patient. We now improved our section 2.3 with this information: “For each patient, out of the 1-2 hours available recording we selected 20 minutes with the highest number of visually marked spikes during sleep.”

12. We recommend to remove the word 'innovative' from the text as this is an opinionated term.

RESPONSE: Following the reviewer suggestion, we have now removed the word ‘innovative’ from the manuscript.
