# Supplementary material for: Data-driven approach for the delineation of the irritative zone in epilepsy in MEG
Source: PLoS One. 2022 Oct 25;17(10):e0275063. doi: 10.1371/journal.pone.0275063 (PMC9595543; doi:10.1371/journal.pone.0275063)
Supplement: Attachment — Submitted filename: Response to Reviewers.docx [file pone.0275063.s005.docx]

Response to Reviewers

We thank the reviewers for their valuable comments.

As requested, we address the concern of misinterpreting the results of the paper by Plummer et al.

Reviewer comment (all text below is from the Editor and Reviewers letter).

“””

Reviewer #2: In relation to this response:

"Further to Point 7, to argue that results would be closer to the accuracy of the VIS

results closer to the take-off is conjectural. Why not run a further analysis closer to take-off (eg.

half-way between take-off and the 50% rising phase?

RESPONSE: We share the reviewer’s interest in the information contained in the takeoff

latency and we tested the possibility to extract valuable information from earlier

latencies of the ascending slope. We illustrate the results of this analysis in the figure,

where we show stable SNR for latencies selected at 50% slope and later, while between

take off and 50% slope the reconstruction does not provide valuable clinical information.

We find our results in line with Plummer et al., 2019.

(I have read over this paper (by Plummer et al) which actually does find valuable clinical information between take off and the 50% slope (so not in line with the paper by Plummer et al in this respect)

We provide the outcome of our analysis in Figure S2 in Supplementary Material, and

refer to it in the Methods, section 2.6.3:

“An activation map was computed for each atom at two latencies identified as PEAK, i.e.

the latency of maximum amplitude of the sharp deflection of the spike, and SLOPE,

identified as the latency preceding the PEAK where the activity is still above baseline

and the spatial pattern provides a distinct focus (see also supplementary material, figure

S2)”

Plummer, Chris, Simon J. Vogrin, William P. Woods, Michael A. Murphy, Mark J. Cook, and

David T.J. Liley. 2019. “Interictal and Ictal Source Localization for Epilepsy Surgery Using High-

Density EEG with MEG: A Prospective Long-Term Study.” Brain 142 (4): 932–51.

<https://doi.org/10.1093/brain/awz015>.

“””

OUR RESPONSE: We thank the reviewer for pointing to the difference with Plummer et al. 2019. We know acknowledge this difference in the Discussion session

The consideration of the ascending slope in MEG has proven strong association of the irritative zone with good outcome [(Englot et al. 2015)](https://www.zotero.org/google-docs/?broken=W0CUFa), while recent evidence from high density EEG and MEG identifies the highest accuracy in the earliest resolvable phase of the IED onset [(Plummer et al. 2019)](https://www.zotero.org/google-docs/?Z5Jeg3).

While our focus in this project is the validation of spike detection and clustering, we plan to compare our approach with single event based analysis on a more comprehensive dataset which we are collecting for a follow-up study.
